# Crystal Structure of the C-Terminal Cytoplasmic Domain of Non-Structural Protein 4 from Mouse Hepatitis Virus A59

**DOI:** 10.1371/journal.pone.0006217

**Published:** 2009-07-10

**Authors:** Xiaoling Xu, Zhiyong Lou, Yanlin Ma, Xuehui Chen, Zhangsheng Yang, Xiaohang Tong, Qi Zhao, Yuanyuan Xu, Hongyu Deng, Mark Bartlam, Zihe Rao

**Affiliations:** 1 Laboratory of Structural Biology, Tsinghua University, Beijing, China; 2 National Laboratory of Biomacromolecules, Institute of Biophysics (IBP), Chinese Academy of Sciences, Beijing, China; 3 College of Life Sciences and Tianjin Key Laboratory of Protein Science, Nankai University, Tianjin, China; Karolinska Institutet, Sweden

## Abstract

**Background:**

The replication of coronaviruses takes place on cytoplasmic double membrane vesicles (DMVs) originating in the endoplasmic reticulum (ER). Three trans-membrane non-structural proteins, nsp3, nsp4 and nsp6, are understood to be membrane anchors of the coronavirus replication complex. Nsp4 is localized to the ER membrane when expressed alone but is recruited into the replication complex in infected cells. It is revealed to contain four trans-membrane regions and its N- and C-termini are exposed to the cytosol.

**Methodology/Principal Findings:**

We have determined the crystal structures of the C-terminal hydrophilic domain of nsp4 (nsp4C) from MHV strain A59 and a C425S site-directed mutant. The highly conserved 89 amino acid region from T408 to Q496 is shown to possess a new fold. The wild-type (WT) structure features two monomers linked by a Cys425-Cys425 disulfide bond in one asymmetric unit. The monomers are arranged with their N- and C-termini in opposite orientations to form an “open” conformation. Mutation of Cys425 to Ser did not affect the monomer structure, although the mutant dimer adopts strikingly different conformations by crystal packing, with the cross-linked C-termini and parallel N-termini of two monomers forming a “closed” conformation. The WT nsp4C exists as a dimer in solution and can dissociate easily into monomers in a reducing environment.

**Conclusions/Significance:**

As nsp4C is exposed in the reducing cytosol, the monomer of nsp4C should be physiological. This structure may serve as a basis for further functional studies of nsp4.

## Introduction

Coronaviruses are positive-strand RNA viruses, characterized by the largest RNA genomes and a nested set of subgenomic mRNA during transcription [1], [2], [3]. MHV strain A59 is an extensively studied group II coronavirus [4], [5] that causes enteritis, hepatitis, and central nervous system demyelination in susceptible mice [6], [7]. Translation of the MHV-A59 genome yields 16 non-structural proteins (nsps), which are encoded by two large overlapping open reading frames (ORF) termed ORF1a and ORF1b. Expression of ORF1b requires a (−1) ribosomal frame-shift to yield two large polyproteins, pp1a (495 kDa) and pp1ab (803 kDa) [8], [9]. The pp1a and pp1ab polyproteins are co-translationally and post-translationally processed by two papain-like proteases domains in nsp3, named PLP1 and PLP2, and the main protease nsp5 (Mpro) into 16 non-structural proteins [10], [11], [12], [13]. These non-structural proteins are proposed to form a membrane-anchored replication complex for RNA replication and transcription [14], [15].

Association of RNA replication with the cytoplasmic membranes of infected cells is a typical feature of all characterized positive-strand RNA viruses to date [16], [17], [18], [19]. Van der Meer and colleagues demonstrated that MHV RNA synthesis and genome replication occur on late endosomal or lysosomal membranes [20]. In contrast, Gosert and coworkers reported that the MHV replication complex is localized to the cellular double membrane vesicles (DMVs); the RNA synthesis, replication and subgenomic mRNA transcription also take place in the cytoplasmic DMVs [21]. It was further demonstrated that the autophagic pathway is required for formation of virus induced double membrane vesicles, and formation of DMVs significantly enhances the efficiency of MHV replication [22]. An electron microscopy study also revealed the involvement of DMVs in formation of the SARS coronavirus replication complex; the DMVs were labeled by several nsps and viral RNA, and the endoplasmic reticulum (ER) was identified as the most likely membrane donor of SARS-CoV induced membrane structures [14]. Thus, it is generally believed that coronavirus replication takes places on the cytoplasmic DMVs, the membranes of which originate from the ER [23]. However, the mechanism of interaction between the replication complex and membranes are unclear and require further investigation.

Studies on a variety of RNA viruses have revealed that replication complexes are associated with cellular membranes by hydrophobic domains from non-structural proteins. Of the 16 coronavirus non-structural proteins, only nsp3, nsp4 and nsp6 are known to possess trans-membrane domains [2], [8], [24], [25], [26] and are proposed to be involved in the interaction of replication complex with the cytoplasmic membranes, either as membrane anchors or as scaffolds for the replication complex [26], [27]. Membrane topology studies concerning SARS and MHV hydrophobic domains of non-structural proteins have revealed that nsp3, nsp4 and nsp6 all have a Nendo/Cendo topology, with both the amino and carboxy terminus exposing to the cytoplasm; furthermore, two of the three hydrophobic domains of nsp3, four hydrophobic domains of nsp4 and six of the seven hydrophobic domains of nsp6 are membrane spanning [26]. These trans-membrane domains comprise of the majority part of the polyprotein pp1a, which are generally believed to mediate the assembly and targeting of replication complex to cytoplasmic membranes [28], [29], [30], while the ORF1b-encoded proteins (nsp12–nsp16) are directly involved in coronavirus RNA replication and transcription [15], [31].

MHV nsp4 was first biochemically identified as an integral membrane protein by the evidence that it is pelleted with detergent Triton_X114 extraction fraction of cell lysates, it is processed by PLP2 at amino terminal and nsp5 at carboxy terminal from a p150 precursor [21]. MHV nsp4 was localized to the endoplasmic reticulum (ER) membrane when expressed alone and recruited to the replication complex in infected cells, its amino and carboxy termini are exposed to the cytosol, while the N-glycosylated region is located between the first and second TM regions and faces the ER luminal side [25]. Substitution of the MHV nsp4 glycosylation site N237 to alanine is lethal for virus replication, while the temperature sensitive mutant N258T virus leads to a dramatic reduction in DMVs assembly at non-permissive temperature, indicating a critical role for nsp4 in coronavirus DMVs assembly [32]. Further virus recovery analysis of several deletion mutants of MHV nsp4 revealed that it is required for viral replication: the putative TMs1-3 and specific charged residues are essential for productive virus infection, while TM4 and the carboxy terminal amino acids K398-T492 are dispensable [33]. It is evident that nsp4 plays important role in coronavirus replication and DMVs formation, and that the TM regions of nsp4 are involved in association of the coronavirus replication complex with cellular membranes. However, our understanding of the functional role of nsp4 is still at an early stage, and there has been no structural characterization of the coronavirus nsp4 to date.

More recently, strong immunolabeling of the nsp4 C-terminal was observed on the double membrane vesicles from SARS-CoV infected cells [34]. The highly conservation of this fragment among all coronavirus species and its involvement in assembly of the DMVs suggests that this cytoplasmic fragment of nsp4 may play an important role in coronavirus replication complex assembly. Here, we report the crystal structure of the C-terminal hydrophilic domain of nsp4 (nsp4C) from MHV strain A59, together with the structure of a C425S site-directed mutant. The structure covers an 89 amino acid region from T408 to Q496 and is shown to possess a new fold. Two wild-type monomers in one asymmetric unit are linked by a Cys425-Cys425 disulfide bond to form a dimer, which could be observed and easily dissociate in reducing solution *in vitro*. Mutagenesis of Cys425 to Ser broke the disulfide bond, but two mutant monomers interact with each other via their cross-linked C-termini to form a strikingly different “close” conformation compared to the “open” conformation of the wild-type dimer, in which the C-termini of the wild-type monomers are oppositely oriented. Through analysis of these two conformations of the dimer and the cellular localization of nsp4C, we conclude that nsp4C exists as a monomer in the cytosol. This structure may serve as a basis for the functional studies of nsp4 from coronaviruses, thus providing preliminary structural insights into the membrane anchoring of coronavirus nsp4.

## Results

### The nsp4C monomer possesses a novel fold

The crystal of WT nsp4C belongs to space group *C2* and the structure covers amino acid residues from T408–T492, while the C425S mutant belongs to space group *P4_1_2_1_2* and covers residues from T408-L495.

#### The monomer structure of WT nsp4C

**Figure 1:**
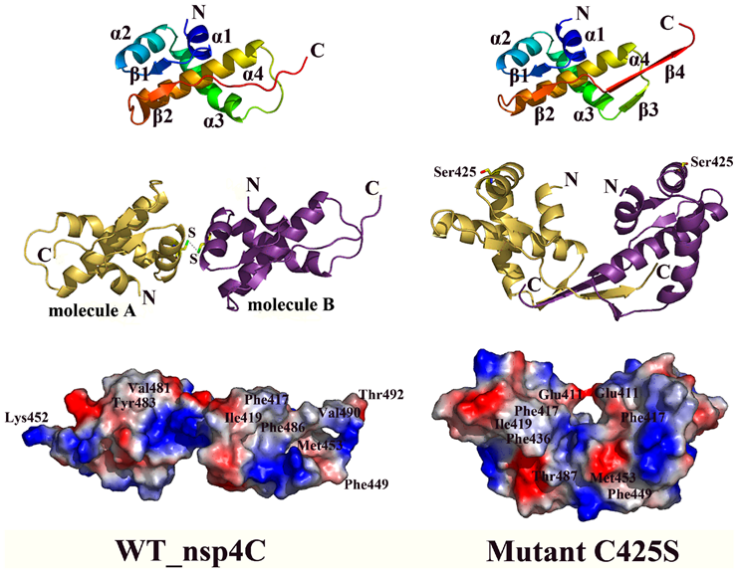
Monomer and dimer representation of the WT nsp4C (T408-Q496) and the C425S mutant. Monomers of the WT and C425S mutant are colored from blue at the N-terminus to red at the C-terminus, secondary elements are labeled. One monomer is colored in magenta, and the other in gold in the dimer. Cys425 forming the disulfide bond in the WT structure and the Serine in the C425S mutant are shown in stick representation; carbon, nitrogen, oxygen, and sulfur atoms are colored in yellow, blue, red, and green, respectively. Surface representation of the electrostatic potential of WT and mutant C425S is at the bottom, specific amino acids are labeled. The figure is drawn by PyMol [47].

The WT nsp4C monomer contains four α-helices (α1–α4) and two short β-strands (β1–β2). The N-terminal starts from a short α-helix (α1, T408–T415), followed by a short β-strand (β1, T416–T420) connecting α1 and another short α-helix (α2, E422–S430), which is connected to a long α-helix (α3, S432–Y446) by a short loop. Notably, a long loop connects helix α3 and another long α-helix (α4, D454–N474); α-helices α3 and α4 point towards each other, and the loop connecting them enables α4 to turn to the back of α3 and connect by a short loop to β-strand β2 (β2, D480–Q484). The strands β2 and β1 are anti-parallel, while β2 is extending further via a long loop to the C-terminus of the molecule (Fig. 1).

**Figure 2:**
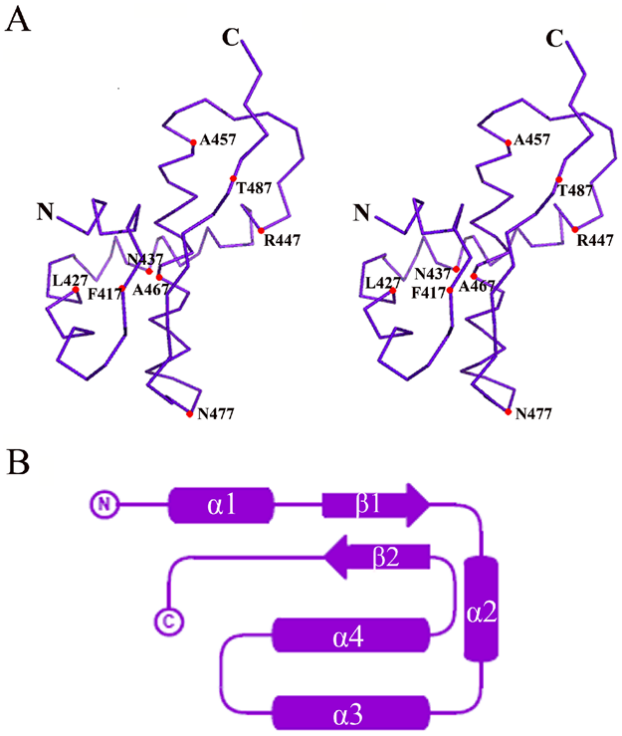
Monomer structure of nsp4C possess a new fold. A. Stereo view of the nsp4C monomer Cα backbone trace. Positions of selected residues and the N- and C-termini are labeled. B. Topology of the nsp4C monomer. β-strands are shown in arrows, and α-helices in cylinders. Fig. 2A was drawn with the programs with PyMol [47] and 2B with TopDraw.

**Figure 3:**
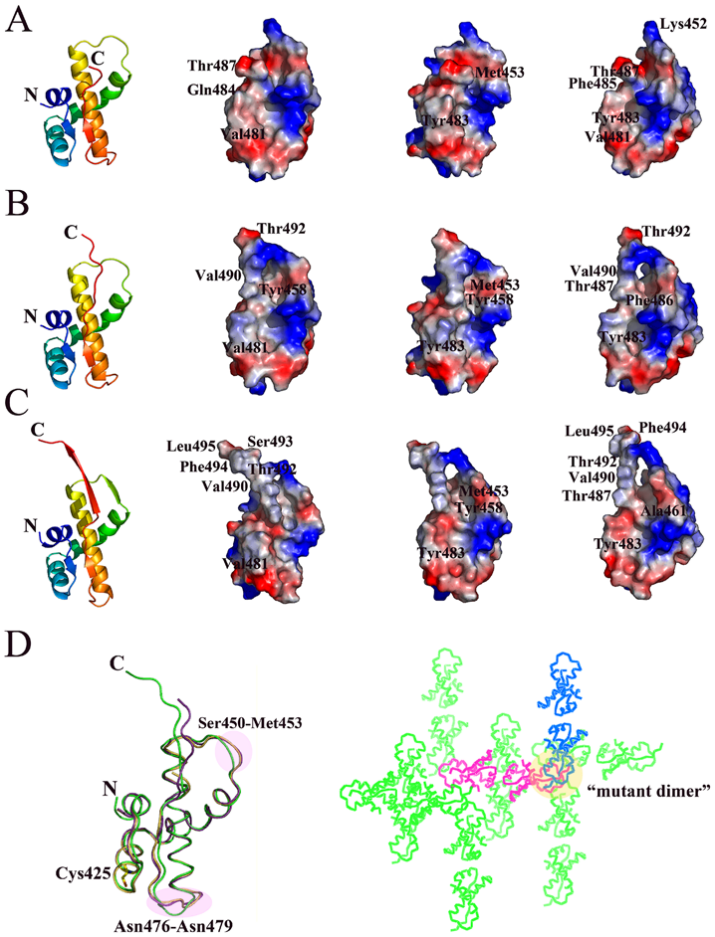
The molecular surface model of the monomer from nsp4C (T408-Q496) WT dimer and mutant C425S. Electrostatic potential is mapped on the surface, with positive charged region colored in blue and negative charged region in red. Both molecules in one asymmetric unit of the WT nsp4C dimer and mutant C425S are shown in three orientations. Fig. 3A and 3B represents molecule A and molecule B of the WT nsp4C dimer respectively; while Fig. 3C represents the monomer of the C425S mutant. Fig. 3D represents the superposition of molecule A (gold) and B (magenta) from WT and the monomer of the C425S mutant (green), Cys425 is shown in stick representation, with the carbon, nitrogen, oxygen, and sulfur atoms colored yellow, blue, red, and green, respectively; and also the crystal packing of WT nsp4C (magenta), symmetry-related molecules are colored in green, and the molecule forming the equivalent of the C425S mutant dimer is colored in blue. Selected amino acids are labeled, and the figure is drawn by PyMol [47].

A DALI search for structural similarity reveals no close homolog to nsp4C in the Protein Data Bank, indicating this structure should have a new fold (Fig. 2A and 2B). The refined WT model contains two nsp4C molecules in one asymmetric unit, termed A and B respectively. The structures of molecule A and B are generally similar with a root mean square deviation (r.m.s.d.) between equivalent Cα atoms of 0.47 Å, although there was a notable lack of electron density for residues S489-Q496 in molecule A and residues S493-Q496 in molecule B. From the crystal packing of WT dimer shown in Fig. 3D, it is evident that C-terminal of molecule B in the dimer (colored in magenta) intersects with the C-terminal of another symmetry-related molecule (colored in blue); the same interaction is not observed in the C-terminal of molecule A. Thus, the interaction with symmetry-related molecules at the C-terminal of molecule B helps to stabilize its conformation and results in detectable electron density from S489-T492. The lack of this interaction in molecule A results in its flexibility and undetectable electron density from S489-Q496.

The electrostatic surface potential of molecules A and B is shown in Fig. 3A and 3B respectively. Notably, the surfaces of both molecule A and B show a hydrophobic cavity formed by residues Ile419, Met453, Thr455, Tyr458, Ala461, Ala462, Gln465, Leu466, Ala469, Phe473, Val481, Leu482, Tyr483, Pro485, Pro486, Thr487 and Ala488. The region from Ala488 to Thr492 bends over the hydrophobic patch located in the loop connecting the helices α3 and α4 to form a hole on the surface. Amino acids located in the Ala488 -Thr492 and Ser450-Met452 loops are critical in forming the hole. The positively charged amino acids Tyr443 and Arg447 in α2, and Lys467 in α3, form a positive charged region around this hydrophobic cavity.

#### The monomer structure of nsp4C mutant C425S

Both WT nsp4C and the C425S mutant contain two monomers in one asymmetric unit, and the conformation of the WT and mutant monomers are quite similar, with a root mean square deviation (r.m.s.d.) between equivalent Cα atoms of less than 1.0 Å. The major differences arise from three regions in the monomer (Fig. 3D). The first is the loop from Ser449 to Met452 which forms an additional β-strand β3 in the C425S mutant; interaction of this region with the C-terminal of the monomer in the mutant dimer produces this deviation. The second region is located between Asn476–Asn479, which is formed by interaction with symmetry related molecules as a result of crystal packing (Fig. 3D). The third occurs in the C-termini of the monomers, where an additional β-strand β4 in the mutant monomer is observed with detectable electron density from Ser493 to Leu495, while the equivalent region of the wild-type monomer exists as a long loop.

Thus, the mutant C425S monomer structure contains four α-helices (α1–α4) and four β-strands (β1–β4). The conformation of the four α-helices and β-strands β1–β2 are identical to WT, while a short β-strand (β3, S449–M452) between α3 and α4 and a long β-strand β4 (β4, T487-S493) at the C-terminal are formed for stability of the dimer during the crystal packing. The electrostatic surface potential of the mutant monomer is similar to WT nsp4C but with the addition of an extended hydrophobic surface formed by the elongated C-terminal, the hydrophobic cavity is similar in both the WT and C425S mutant structures, and the Ala488-Leu495 (β4) and Ser450-Met452 (β3) loops are critical in forming the hole in the surface of mutant monomer (Fig. 3C). It is also likely that the C-terminal is essential for stability both in the WT nsp4C and C425S mutant, since the expression of a ΔF484-Q496 deletion mutant has very low yield compared to the WT nsp4C protein.

### Two conformations of the nsp4C dimer

Both the WT and mutant C425S contain two monomers in one asymmetric unit. However, the conformations of these two dimers are strikingly different.

#### The dimer conformation of WT nsp4C

In the WT nsp4C structure, two monomers in one asymmetric unit are linked by a 2.0 Å Cys425-Cys425 disulfide bond to form a dimer (Fig. 1). The C-terminal of nsp4 is exposed to the cytosol, suggesting that the nsp4C should bind to the ER membrane via its N-terminal. Notably, the N-termini of the two molecules are not aligned in the WT nsp4C dimer. Instead, they are projected in different orientations, with one molecule rotated by about 150° around the y-axis relative to the other molecule (Fig. 1). The N-terminal amino acids Cys425, Lys421, Glu422, Lys426, and Asn429 are located in the contact surface between the two monomers. The hydrophobic cavity of WT nsp4C is exposed on the dimer surface, and the C-termini of the two monomers extend in different orientations. We propose that the surface properties of the WT nsp4C dimer are representative of an “open” conformation given that its positive, negative and hydrophobic areas are all exposed (Fig. 1). This conformation is in conflict with the hypothesis that nsp4C binds to the membrane as a dimer via their N-termini; it is rational only if nsp4C binds the membrane as a monomer.

#### The dimer conformation of mutant C425S

Two monomers of the mutant C425S also form a dimer in the asymmetric unit, with their N-termini in parallel and in the same orientation. The C-terminal β4 strands of the two monomers are cross-linked and form anti-parallel β-sheets together with β3 at the bottom of the dimer (Fig. 1).

Ser425 from each monomer in the mutant dimer is oriented on the outside of the dimer, far away from intersecting C-termini of the dimer (Fig. 1). Thus, mutating Cys425 to Ser does not directly influence the interaction between the C-termini of these two monomers. The mutant C425S dimer could be obtained by crystallographic symmetry of the WT nsp4C structure (Fig 3D). In WT nsp4C, a disulfide bond at Cys425 causes the C-termini of the two monomers to extend in different orientations, and thus resulting in flexibility and undetectable electron density differences at the C-termini of the monomers. In the case of the C425S mutant, the disulfide bond is dissociated and the dimer adopts a more stable conformation by crystal packing: the flexible C-termini of two monomers are stabilized by hydrogen bond interactions between the amino acids in the Ala488-Thr492 (β4) and Ser450-Met452 (β3) loops, which form stable anti-parallel β-sheets at the bottom of the dimer, resulting in clear electron density from residues Ser489-Leu495.

The electrostatic surface potential of the dimer differs from that of WT nsp4C. The positive and negative charges are distributed equally on the surface, and Glu411 from each monomer are close to each other to form a hole in the middle of the dimer. The intersecting C-termini of the monomers result in a “closed” conformation of the mutant dimer (Fig. 1). The parallel N-termini of the two monomers are much more convenient for the binding of nsp4C to the membrane as a dimer, but this conformation is formed by mutagenesis and it could also be identified in the crystal packing of WT nsp4C (Fig.3D). Thus the conformation of mutant C425S dimer is not likely to be physiological but instead an artifact of crystallization.

### Switch between the nsp4C monomer and dimer in solution

**Figure 4:**
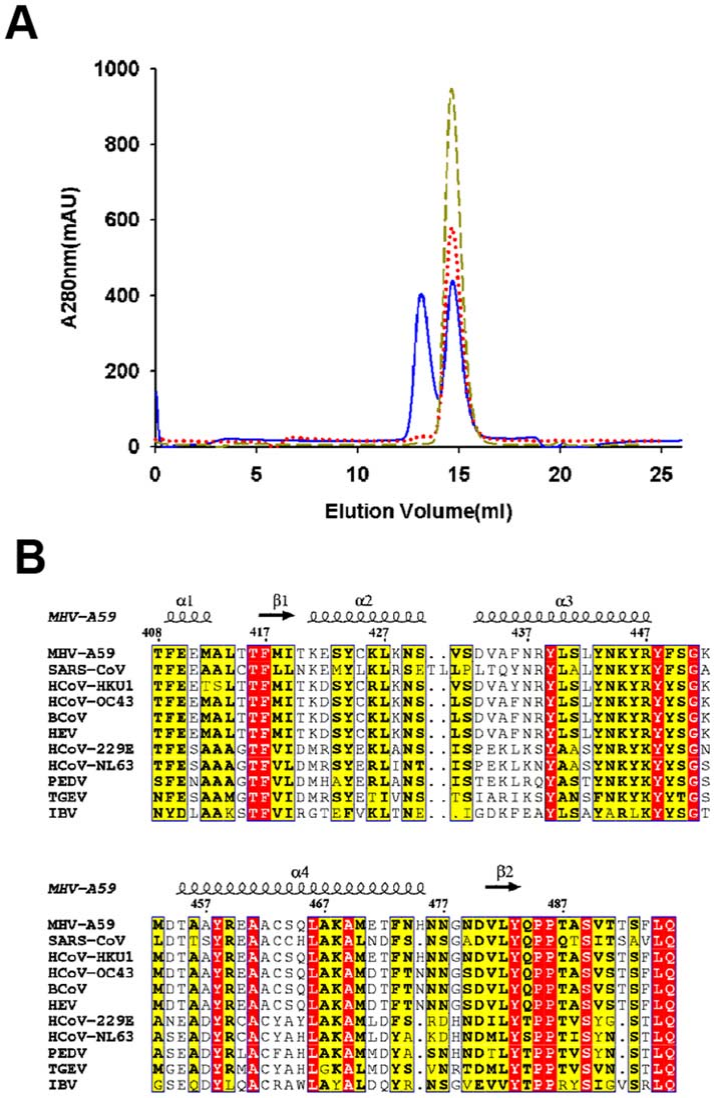
A. Gel filtration analyses of wild-type nsp4C (T408-Q496) and the C425S mutant. WT, WT with 1mM DTT and C425S were separately loaded in the same quantity and eluted at 0.5 mL min^−1^ with a buffer containing 50 mM Tris-HCl (pH 8.5), 150 mM NaCl. WT measurements are represented in solid blue line, C425S in medium dashed dark yellow line, and WT containing 1mM DTT in dotted red line. WT was eluted in two peaks corresponding to dimer and monomer respectively; in contrast, C425S and WT containing 1 mM DTT were dominated only by the monomer. B Multiple sequence alignment of MHV-A59 nsp4C (T408-Q496) with the representatives from all three groups of the genus Coronavirus. Key: MHV-A59, Mouse Hepatitis Virus strain A59, NP_001012459; SARS-CoV, Severe Acute Respiratory Syndrome, NP_904322; HCoV-HKU1, Human Coronavirus HKU1, YP_459935; HCoV-OC43, Human Coronavirus strain OC43, NP_937947; BCoV, Bovine Coronavirus, NP_742170; HEV, Porcine Hemagglutinating Encephalomyelitis Virus, YP_459949; HCoV-229E, Human Coronavirus strain 229E, NP_835358; HCoV-NL63, Human Coronavirus strain NL63, YP_003766; PEDV, Porcine Epidemic Diarrhea Virus, NP_598309; TGEV, Transmissible Gastroenteritis Virus, NP_058422; and IBV, Avian Infectious Bronchiolitis Virus, NP_066134. Secondary structural elements of the crystal structure are shown at the top of the alignment; arrows indicate β-strands, and helical curves denote α(or 310)-helices. Residues highlighted in red are identical among the compared proteins; residues highlighted in yellow are conserved. The alignment was generated using the program ClustalX [48] and drawn with ESPript [49].

The dimerization of nsp4C is also detected by gel filtration (Fig. 4A). Elution of purified WT nsp4C protein through a Superdex75 column in buffer containing 50 mM Tris-Cl, pH 8.5 and 300 mM NaCl yields two distinct 280 nm absorption peaks: the first peak appears at 13.1 ml and the second at 14.67 ml. According to the profiles of standard marker proteins such as aprotinin (MW: 6,512 Da), RNaseA (MW: 13,700 Da), albumin egg (MW: 45,000 Da) and BSA (MW: 67,000 Da) on the same column and under the same buffer conditions, these two peaks correspond to the dimer and monomer of nsp4C, respectively. While the nsp4C sample in the presence of the reducing agent DTT exhibits a different elution profile under the same conditions, it yields only one 280 nm absorption peak at 14.63 ml, which corresponds to the nsp4C monomer. Furthermore, the elution profile of the C425S point mutant was identical to the profile of nsp4C with DTT, with only a single absorption peak appearing at 14.64 ml and corresponding to the monomer. The reducing agent β-ME also works to reduce this disulfide bond. The dimer exists even in SDS-PAGE analysis under non-reducing conditions (data not shown).

The WT nsp4C exists both as dimer and monomer *in vitro*, and the dimer can easily dissociate into monomers under reducing environment; mutation of Cys425 to serine disrupts the disulfide bond and only yields monomers. Our WT nsp4C crystal was obtained under non-reducing solutions, so it is considerably easier for two nsp4C monomers to form a disulfide bond in the crystal. The C425S mutant dimer was obtained by mutagenesis and crystal packing, and is unlikely to represent a physiological state. No enzymatic activity or biochemical studies have been reported for nsp4 to date, so further studies are required to confirm whether or not the WT nsp4C dimer is physiological.

## Discussion

### Nsp4C exists as a monomer in the cytosol

Topology studies of MHV nsp4 indicate that the N-terminal of nsp4C is connected to the ER membrane by about 8 amino acids, -TEVRSDG-, when nsp4 is initially processed from the p150 precursor; the region spanning residues T408 to Q496 is located in the cellular cytosol. The cytosol has long been regarded as a reducing cellular compartment in which the reduced state of cysteine in proteins is largely favored; the disulfide bond is generally formed in the more oxidizing ER lumen rather than the reducing cytosol [35], [36]. Furthermore, our results confirm that the nsp4C dimer can readily switch to a monomer in a reducing environment, so it is evident that nsp4C may exist as monomer in the reducing environment of the cytosol when it is expressed alone in uninfected cells. Our crystal structure further revealed that the N-termini of the two monomers in the WT nsp4C dimer are not suitably aligned to bind to the ER membrane. And also the dimer is formed in a non-reducing and highly condensed protein solution, and is therefore not representative of the physiological state. Moreover, from the multiple sequence alignment in Fig. 4B, the Cys425 is not a conserved amino acids among the coronavirus nsp4 proteins, thus the dimer is a specific case to MHV nsp4C. Mutation of Cys425 to Ser prevents formation of the disulfide bond, which does not influence the conformation of monomer, so the conformation of monomer is stable and conserved. Thus, the monomer of nsp4C may be physiologically functional when it is bound to the ER membrane in the cytosol.

### Role of nsp4C in coronavirus replication

It was recently reported that the C-terminal fragment from K398 to T492 of nsp4 is dispensable for viral replication [33]. Generally speaking, nsp3 and nsp4 are reported to be inserted into the ER membrane via their hydrophobic sequences [24], [25], while other nsps lacking hydrophobic sequences are believed to localize in the cytoplasm [2]. The trans-membrane domain of nsp3 is sufficient to mediate ER membrane association of the cytosolic protein EGFP [24]. The poliovirus (PV) membrane proteins 2BC and 2C are also dispensable for viral RNA replication, but are exclusively associated with the formation of PV replication vesicles; 2BC recruits the COPII complex to the ER membranes to trigger vesicle formation [37], [38]. Furthermore, topology study revealed that the N-terminal may serve as signal peptide, and that the hydrophilic region between the first and second trans-membrane domain is exposed to the ER lumen and is glycosylated in the ER [25]. Other hydrophilic regions of nsp4 are too short to interact with other proteins, and nsp4C was detected on the double membrane vesicles from SARS-CoV infected cells [34], thus the cytosol exposed C-terminal of nsp4 should be involved in interactions with other viral or host cell proteins in replication.

The surface of the nsp4C crystal structure suggests that the most likely candidate region for protein interaction may be the hydrophobic cavity formed by Ile419, Met453, Thr455, Tyr458, Ala461, Ala462, Gln465, Leu466, Ala469, Phe473, Val481, Leu482 Tyr483, Pro485, Pro486, Thr487 and Ala488. In the WT nsp4C structure, the Ser450-Met452 and Ala488-Thr492 loops are the most flexible regions of the molecule, but form more stable elements β3 and β4 respectively in the C425S mutant via interactions with adjacent molecules in the asymmetric unit, suggesting that interaction of these regions with other partner molecules could help to stabilize the nsp4C molecule. SARS coronavirus nsp2 reportedly interacts with nsp4 and nsp6 *in vitro* [39]. However, our Surface Plasmon Resonance (SPR)-based interaction assays of nsp4C with several MHV A59 nsps did not indicate any direct interaction between nsp4C and other nsps (data not shown). Since not all of the MHV ORF-encoded proteins were tested, further interaction studies are required to identify the nsp4C partner protein.

Multiple sequence alignment of MHV-A59 nsp4 with representatives from all three groups of the genus Coronavirus reveals that the membrane topology of nsp4 from all group members is similar. In particular, the C-terminal hydrophilic region from T408 to Q496 is highly conserved among all the coronaviruses (Fig. 4B), indicating that nsp4C may play a similar and conserved role in coronavirus replication. In summary, the crystal structure of MHV-A59 nsp4C reported here provides a preliminary structural description of coronavirus non-structural protein nsp4, further functional and structural studies of nsp4, together with its interaction with other virus and host cell proteins, will certainly boost our understanding the role of nsp4 in coronavirus replication.

## Methods

### Protein expression and purification

Using a multiple sequence alignment of nsp4 from representatives of the genus *Coronaviridae*, together with TMHMM and Tmpred predictions from the primary sequence, we successfully designed the construct of this highly conserved hydrophilic domain of MHV-A59 nsp4. The gene fragment encoding the MHV-A59 nsp4 carboxy terminal domain (nsp4C) spanning T3252 to Q3328 of pp1a was cloned from the virus genomic cDNA library by polymerase chain reaction (PCR) into the pGEX-6p-1 vector (GE Healthcare), and the resulting plasmid was transformed into *Escherichia coli* BL21 (DE3) cells. The recombinant glutathione transferase (GST) fusion protein, GST−nsp4C, was purified by GST-glutathione affinity chromatography. The cells were disrupted by sonication in 50 mM Tris-Cl pH 8.5, 200 mM NaCl and 2 mM DTT, and the cell lysates were centrifuged at 15,000 rpm for 30 min. The soluble fraction was then applied on a GST-affinity chromatography column for purification. The GST tag was removed by PreScission protease (GE Healthcare), leading to five additional residues (GPLGS) at the N-terminus. The protein sample purified by affinity chromatography was further purified by anion ion-exchange chromatography on a ResourceQ column with an elution buffer containing 50 mM Tris-Cl, pH 8.5 and a gradient concentration of NaCl to 1 M. The flow through fraction from Resource Q column was collected for gel filtration purification on a Superdex75 column in buffer containing 50 mM Tris-Cl pH 8.5 and 150 mM NaCl. A C425S site-directed mutant was constructed by overlapping extension PCR [40], and was then expressed, and purified using the same protocol.

### Crystallization

MHV-A59 nsp4C was crystallized by the hanging drop vapor diffusion method with a 2 µL drop volume at 18°C. 1.0 µL nsp4C at 3 mg ml^−1^ was added to the same volume of reservoir solution (10% PEG MME550, 0.1 M glycine, pH 9.0, and 0.01 M zinc sulfate), producing plate like crystals belonging to the space group of *C2*. Crystals of the selenomethionyl (Se-Met) substituted nsp4C were obtained from the same conditions to solve the phase problem. The Se-Met crystals shared the same space group and similar unit cell dimensions as the wild-type (WT) crystals. The WT and Se-Met derivative crystals of nsp4C were highly susceptible to nucleation and twinning. Crystals of the C425S mutant belonging to the space group *P4_1_2_1_2* were obtained under the condition of 26% PEG MME550, 0.1 M glycine pH 9.0, and 0.01 M zinc sulfate with a protein concentration at 6 mg ml^−1^.

### Data collection

The crystals were cryoprotected in a solution containing the reservoir solution with 15% glycerol and flash-cooled with liquid nitrogen. A 2.0 Å resolution data set of native nsp4C was collected at 100 K on beamline BL-5A of the Photon Factory in Japan using an ADSC Q315 detector. The crystal form belongs to the cubic space group *C2* (*a* = 86.86, *b* = 52.55 Å, *c* = 53.87 Å, α = γ = 90°, β = 116°) with two molecules in one asymmetric unit and a Matthews coefficient V_M_ of 2.6 Å^3^ Da^−1^, corresponding to a solvent content of 57% [41]. The Se-Met derivative data of nsp4C were collected at BSRF (Beijing Synchrotron Radiation Facility) at 2.4 Å resolution using a Mar CCD detector. The Se-Met crystals shared the same space group and similar unit cell parameters with the native crystals. Data for the C425S mutant were collected to 2.4 Å resolution on beamline BL-17A of the Photon Factory in Japan using an ADSC Q270 detector. The crystal form belongs to the primitive tetragonal space group *P4_1_2_1_2* (*a* = *b* = 54.79 Å, *c* = 97.55 Å, α = β = γ = 90°). Processing of all diffraction images and scaling of the integrated intensities were performed using the HKL2000 software package [42].

### Structure determination

The structure of nsp4C was solved by the single-wavelength anomalous dispersion (SAD) method from a Se-Met derivative crystal. Three out of the four expected selenium atoms in one molecule were located and initial phases were calculated by the program SOLVE [43]. After density modification (solvent flipping) using the program RESOLVE, a clearly interpretable electron density map was calculated. The model of nsp4C was then manually built and refined using the programs O [44] and CNS [45]. The crystal structure was refined at 2.0 Å resolution to a final *R_work_* of 22.1% and *R_free_* of 26.4%. The structure of the C425S mutant was solved by molecular replacement (MR). A model was obtained with the program PHASER from the CCP4 package [46], then further refined and rebuilt with CNS and O respectively. The crystal structure of the C425S mutant was refined at 2.4 Å resolution to a final *R_work_* of 22.7% and *R_free_* of 26.6%. Refinement statistics are detailed in Table 1.

**Table 1:** Data collection and refinement statistics.

Parameters	Native nsp4C	Se-nsp4C	Mutant C425S
**Data collection statistics**			
Cell parameters	*a* = 86.86 Å	*a* = 85.26 Å	*a* = *b* = 54.79 Å
	*b* = 52.55 Å	*b* = 51.56 Å	
	*c* = 53.87 Å	*c* = 52.67 Å	*c* = 97.55 Å
	α = γ = 90°, β = 116°	α = γ = 90°, β = 116°	α = β = γ = 90°,
Space group	*C2*	*C2*	*P4_1_2_1_2*
Wavelength used (Å)	1.5418	0.9798	1.5418
Resolution (Å)	50 (2.07)^c^– 2.00	50 (2.5)^c^– 2.4	50 (2.49)^c^– 2.4
No. of all reflections	138870	52828	68486
No. of unique reflections	14589	7733	5920
Completeness (%)	98.6 (89.4)	94.2 (81.9)	94.2 (70.9)
Average I/σ(I)	15.0 (6.1)	10.8 (5.8)	11.7 (2.3)
R_merge_ a (%)	5.6 (20.2)	7.8 (19.9)	7.8 (31.0)
**Refinement statistics**			
No. of reflections used (σ(F)>0)	14364		5697
No. of reflections in test set	718		319
R_work_ b (%)	22.1		22.7
R_free_ b (%)	26.4		26.6
r.m.s.d. bond distance (Å)	0.007		0.010
r.m.s.d. bond angle (°)	1.24		1.58
Average B-value (Å^2^)	40.6		54.7
Ramachandran plot (excluding Pro & Gly)			
Res. in most favored regions	143 (94.1%)		151 (91.0%)
Res. in additionally allowed regions	8 (5.3%)		13 (7.8%)
Res. in generously allowed regions	1 (0.7%)		2 (1.2%)

a *R_merge_* = Σ_h_Σ_l_ | I_ih_−<I_h_> |/Σ_h_Σ_I_ <I_h_>, where <I_h_> is the mean of the observations I_ih_ of reflection h.

b *R_work_* = Σ( ||F_p_(obs)|−|F_p_(calc)||)/Σ|F_p_(obs)|; *R_free_*  = R factor for a selected subset (5%) of the reflections that was not included in prior refinement calculations.

c Numbers in parentheses are corresponding values for the highest resolution shell.
